# *Lutzomyia longipalpis* TGF-β Has a Role in *Leishmania infantum chagasi* Survival in the Vector

**DOI:** 10.3389/fcimb.2019.00071

**Published:** 2019-03-27

**Authors:** Tatiana Di-Blasi, Erich Loza Telleria, Christiane Marques, Rodrigo de Macedo Couto, Monique da Silva-Neves, Magdalena Jancarova, Petr Volf, Antonio Jorge Tempone, Yara Maria Traub-Csekö

**Affiliations:** ^1^Laboratório de Biologia Molecular de Parasitas e Vetores, Instituto Oswaldo Cruz, Rio de Janeiro, Brazil; ^2^Parasitology Department, Faculty of Science, Charles University, Prague, Czechia

**Keywords:** *Lutzomyia longipalpis*, *Leishmania*, vector-parasite interaction, innate immunity, TGF-β, activin

## Abstract

Despite the increasing number of studies concerning insect immunity, *Lutzomyia longipalpis* immune responses in the presence of *Leishmania infantum chagasi* infection has not been widely investigated. The few available studies analyzed the role of the Toll and IMD pathways involved in response against *Leishmania* and microbial infections. Nevertheless, effector molecules responsible for controlling sand fly infections have not been identified. In the present study we investigated the role a signal transduction pathway, the Transforming Growth Factor-beta (TGF-β) pathway, on the interrelation between *L. longipalpis* and *L. i. chagasi*. We identified an *L. longipalpis* homolog belonging to the multifunctional cytokine TGF-β gene family (*LlTGF*-β), which is closely related to the activin/inhibin subfamily and potentially involved in responses to infections. We investigated this gene expression through the insect development and in adult flies infected with *L. i. chagasi*. Our results showed that *LlTGF*-β was expressed in all *L. longipalpis* developmental stages and was upregulated at the third day post *L. i. chagasi* infection, when protein levels were also higher as compared to uninfected insects. At this point blood digestion is finished and parasites are in close contact with the insect gut. In addition, we investigated the role of LlTGF-β on *L. longipalpis* infection by *L. i. chagasi* using either gene silencing by RNAi or pathway inactivation by addition of the TGF-β receptor inhibitor SB431542. The blockage of the LlTGF-β pathway increased significantly antimicrobial peptides expression and nitric oxide levels in the insect gut, as expected. Both methods led to a decreased *L. i. chagasi* infection. Our results show that inactivation of the *L. longipalpis* TGF-β signal transduction pathway reduce *L. i. chagasi* survival, therefore suggesting that under natural conditions the parasite benefits from the insect LlTGF-β pathway, as already seen in *Plamodium* infection of mosquitoes.

## Introduction

Leishmaniasis is a serious public health concern, affecting millions of people every year. Leishmaniasis is caused by parasites from the genus *Leishmania*. These parasites are transmitted in the New and Old World by phlebotomine sand flies belonging to the *Lutzomyia* and *Phlebotomus* genera, respectively. When a female sand fly feeds on an infected vertebrate host, macrophages containing amastigote parasites are ingested. Inside the insect gut, the aflagellated immotile rounded parasites transform into flagellated motile promastigotes that multiply and then migrate to the anterior midgut (Lainson and Rangel, 2005). To prevent being eliminated these parasites must resist the blood digestion process (Pruzinova et al., 2018) and adhere to the midgut epithelium prior to insect defecation (Wilson et al., 2010). The presence of *Leishmania* parasites in the insect gut is not harmless to the vector. For instance, parasites secrete a chitinolytic enzyme that attack and damage the sand fly stomodeal valve, leading to the regurgitation of the gut content at the bite site (Rogers et al., 2008). The intricate interactions among *Leishmania* parasites, gut microbioma and the insect immunity are complex, and evolved to avoid the harmful consequences of an eventual uncontrolled microbial or leishmanial growth inside the insect gut (Sant'Anna et al., 2014; Kelly et al., 2017;Telleria et al., 2018).

Most of the knowledge on insect immune responses was acquired from *Drosophila* studies. Pathogens in the insect midgut elicit immune responses that have evolved to eliminate or control infections. These immune responses initiate when the insect innate immune system recognizes the invader. This recognition occurs when pathogen-associated molecular patterns (PAMPs) binds to host-derived pattern recognition receptors (PPRs), leading to the activation of signal transduction pathways. These pathways intensify the immune responses, induce the synthesis of antimicrobial molecules and potentiate effector mechanisms. Among these widely conserved receptor-mediated immune mechanisms, Toll, IMD, and Jak-Stat pathways are the most studied in insects. They are regulators and mediators of insect humoral and cellular immune responses, with intracellular negative regulators that keep these pathways under control (reviewed in Lemaitre and Hoffmann, 2007; Kleino and Silverman, 2014; Lindsay and Wasserman, 2014; Myllymäki and Rämet M, 2014). To date few studies have addressed *L. longipalpis* immunity. The Toll and IMD pathways can be activated in *L. longipalpis* LL5 embryonic cells in response to microbial and *L. i. chagasi* infections (Tinoco-Nunes et al., 2016). Bacterial challenges in *L. longipalpis* larvae leads to a positive modulation of Pirk gene, a suppressor of the IMD pathway at the signal transduction level (Heerman et al., 2015). In adult *L. longipalpis* the RNAi-mediated gene silencing of the IMD pathway repressor Caspar decreased *Leishmania* survival (Telleria et al., 2012). Furthermore the expression of a sand fly defensin was upregulated after artificial infection with different bacteria (Boulanger et al., 2004; Telleria et al., 2013). In insects another important strategy for infection control is the gut environment oxidative stress modulation. The oxidative stress is a consequence of aerobic processes that lead to the production of reactive oxygen species (ROS) such as superoxide, hydrogen peroxide, and nitric oxide (NO), among others (reviewed in Murphy et al., 2011, Kodrik et al., 2015). While aerobic processes occur autonomously in many organisms, some stimuli during development and pathogen challenges can induce ROS production as a result of growth factor receptors and activation of cytokines such as mitogen, integrin and wingless (reviewed in Covarrubias et al., 2008).

The Transforming Growth Factor-beta (TGF-β) pathway, a highly conserved signal transduction pathway in animals, has received very little attention in insects. This cytokine superfamily comprises more than 40 members, grouped into subfamilies according to structure or function: TGF-β *stritu sensu*, bone morphogenic protein (BMP), decapentaplegic (Dpp), Mullerian inhibiting substance, and activin/inhibin (Massague, 1990). These cytokines are involved in several aspects of animal biology including embryonic development, organogenesis, stress responses, and immune modulation (Huminiecki et al., 2009; Massague, 2012; Chen and Ten Dijke, 2016; Morikawa et al., 2016; Mullen and Wrana, 2017). Activins and BMPs are key regulators of immune response having pro- and anti-inflammatory roles (reviewed in Jones et al., 2004; Aleman-Muench and Soldevila, 2012; Lee et al., 2012; Spottiswoode et al., 2017). Activins are secreted in stimulated immune cells and they can act as immune response activator or repressor depending on the which cell type is stimulated (Ogawa and Funaba, 2011).

Although we have some information regarding immunity effectors in sand flies, there is little information on the cytokine-like molecules possibly involved in their regulation. In the present work, we characterized a *L. longipalpis TGF*-β gene (*LlTGF*-β). We also investigated the involvement of *LlTGF*-β in *L. i. chagasi* infection. The blockage of this signal transduction pathway revealed an effect on insect immune related molecules and NO production with consequences to *L. i chagasi* infection.

## Materials and Methods

### Insects

Sand flies were obtained from a laboratory colony of *L. longipalpis* established from sand flies caught in Jacobina (Bahia, Brazil). Insects were fed on 50–70 % sucrose *ad libitum* and females were blood fed on anesthetized hamsters or mice once a week. Females 2–3 day old were used on experimental procedures. All procedures involving animals were performed in accordance with the Brazilian Ethics Committee for Animal Use at FIOCRUZ (CEUA L-016/2018).

### Artificial Infection With *Leishmania*

*L. i. chagasi* (MHOM/BR/1974/PP75) were cultured in M199 medium, pH 7.4, supplemented with 10% fetal bovine serum and collected at exponential growth phase, washed with PBS, and resuspended in inactivated rabbit blood at 10^7^ parasites/mL. Sand flies were infected with promastigotes for practical reasons, since there is evidence that infections initiated with *Leishmania* promastigotes, axenic amastigotes or macrophage-derived amastigotes yield to equally successful infections of sand flies (Freitas et al., 2012; Sadlova et al., 2017). Insects were fed on infective blood through chick skin using a Hemotek artificial feeder. As control group, insects were fed on blood.

For the LlTGF-β receptor inhibition assays, the flies were fed with blood containing *L. i. chagasi* and 10 μM of the TGF-β receptor inhibitor SB431542 (TOCRYS Biosciences). As control the flies were fed with the same volume of the vehicle dimethyl sulfoxide (DMSO) added to the infective blood meal. For these experiments, pools of 10 fully engorged females were collected at 24, 72, and 144 h post infection.

### *LlTGF-β* Gene Identification

Partial *L. longipalpis TGF*-β gene (*LlTGF*-β) sequence was identified through the screening of a *L. longipalpis* EST library. Sequence identity was determined by similarity using the blastx search tool (Altschul et al., 1990) against the NCBI GenBank. Full cDNA sequence was obtained by PCR using an existing cDNA library (Ramalho-Ortigao et al., 2001) with a *LlTGF*-β internal primers (LlTGFblibrary1-R or LlTGFblibrary2-R) and cDNA library plasmid primer (T3) (Table 1). Phylogeny analysis of *LlTGF*-β was performed utilizing Mega 6.06 software, using Maximum Likelihood statistical method with bootstrap method with 500 replicates as phylogeny test. The mutation model used was Jones-Taylor-Thornton with Gamma distribution with invariant sites, and 5 rate categories was chosen according to MEGA 6.06 best mutation model analysis.

**Table 1:** Primers.

**Primer name**	**Sequence**
LlTGFblibrary1-R	GCAGTTGATGTTCCGC
LlTGFblibrary2-R	CTGCGTAATCTTCCCGTAGT
T3	AATTAACCCTCACTAAAGGG
LlTGFb-F	CTCTTCTACTTGGACAGCAA
LlTGFb-R	CATACAGCCGCATCCTTC
LlRP49-F	GACCGATATGCCAAGCTAAAGCA
LlRP49-R	GGGGAGCATGTGGCGTGTCTT
LlHistone-F	GAAAAGCAGGCAAACACTCC
LlHistone-R	GAAGGATGGGTGGAAAGAAG
LlCecropin-F	TGGCAGTCCTGACCACTGGA
LlCecropin-R	CTTCTCCACTGAACGGTGAACG
LlDefensin2-F	ATCCATCCTTTATGCAACCG
LlDefensin2-R	GCCTTTGAGTCGCAGTATCC
LliNOS-F	TGGCTGTCGCAATTTGTGTG
LliNOS-R	CCGCAATGTTCACCTCAACC
dsTGFb-F	TAATACGACTCACTATAGGGGGCTATCATGCCTACTTC
dsTGFb-R	TAATACGACTCACTATAGGGGGCAAAACTTTCTGTGTG
dsBgal-F	TGGCGCCCCTAGATGTGATGGCACCCTGATTGA
dsBgal-R	TGGCGCCCCTAGATGTCATTGCCCAGAGACCAGA
T7-adaptor	CCGTAATACGACTCACTATAGGGTGGCGCCCCTAGATG
Leish-Actin-F	GTCGTCGATAAAGCCGAAGGTGGTT
Leish-Actin-R	TTGGGCCAGACTCGTCGTACTCGCT

### LlTGF-β Double Stranded RNA Synthesis

LlTGF-β cDNA template was amplified by PCR using primers containing the T7 promoter at the 5' end (dsTGFb-F and dsTGFb-R—Table 1). The control dsRNA cDNA template was obtained from β-galactosidase gene amplified from plasmid pGEM T-easy vector (PROMEGA) (Tinoco-Nunes et al., 2016). The PCR products were used as templates for transcription reactions with the Megascript RNAi Kit (Ambion) according to the manufacturer's instructions and subsequently concentrated to 40 μg/μL using a vacuum microcentrifuge concentrator.

### Sandfly Microinjections

*L. longipalpis* female dsRNA microinjection procedure was adapted from Sant'Anna et al. (2008). Shortly, 3 day old sandflies were microinjected with 32 nL of 4 μg/μL ds-LlTGF-β or control dsRNA solution using micro-capillary glass needle coupled to a Nanoject II microinjector (Drummond). Injected flies were artificially blood fed or infected with *Leishmania* as previously described and pools of 5 females were collected at the time-points of 24, 48, and 72 h after feeding.

### RNA Extraction and cDNA Synthesis

Total RNA was extracted with TRIzol reagent (Invitrogen-Life Technologies) according to the manufacturer's protocol, from groups of 5 to 10 fully engorged females collected at 24, 48, 72, and 144 h after blood feeding, after artificial *L. i. chagasi* infection, or after dsRNA microinjection. Total RNA was also extracted from pools of unfed females (0 h), males, eggs, and larvae (L1, L2, L3, and L4 instars). RNA was treated with RQ1 RNase-Free DNase (Promega). First strand cDNA was synthetized with SuperScript III First-Strand Synthesis System for RT-PCR (Invitrogen), oligo dT(16) primer, and up to 1 μg of total RNA.

### Semi-Quantitative PCR of Larvae and Blood Fed or Infected Sand Flies cDNA

Semi-quantitative PCR was carried out using cDNA samples under the following cycling conditions: 96°C/3 min, followed by 25 cycles at 96°C for 45 s, 60°C for 45 s and 72°C for 45 s, and a final extension of 72°C for 5 min. Primers for *LlTGF*-β and constitutive expression controls, histone (Telleria et al., 2007) and RP49 (Tinoco-Nunes et al., 2016), are listed in Table 1. PCR products were separated in 2.0% ethidium bromide-stained agarose gel. The intensity of amplified products was determined by densitometry using the ImageJ software 1.48 software (Schneider et al., 2012). *LlTGF*-β transcription was normalized to histone or RP49 amplification, plotted on GraphPad Prism software (version 6.05—GraphPad Software, Inc). RT-PCR procedures were performed at least 3 times with consistent results. Significance was evaluated by *t*-test and Mann-Whitney post-test, with *p* < 0.05.

### Quantitative PCR Using cDNA From *L. longipalpis* Silenced for *LlTGF-β* or Fed With TGF-β Receptor Inhibitor SB431542

Quantitative PCR was performed using the kit iQTM SYBR Green Supermix (Applied Biosystems) under the following cycling conditions: 96°C/3 min, followed by 42 cycles at 96°C for 30 s, 60°C for 20 s. All experiments were performed using three biological cDNA replicates. The primers used are listed on Table 1. Expression was normalized using the *L. longipalpis* reference gene RP49, relative levels of RNA expressed were calculated using the ΔΔCT method (Schefe et al., 2006) and plotted on GraphPad Prism software. Q-PCR procedures were performed with 3 replicates with consistent results. Significant differences were evaluated by *t*-test and Mann-Whitney post-test, with *p* < 0.05.

### LlTGF-β Recombinant Protein (LlTGF-β*rec*)

A 3′-end fragment (359 bp) of the *LlTGF*-β gene was amplified by PCR using specific primers (TGF-beta-His-F and TGF-beta-His-R), the PCR product was cloned in pGEM-T easy plasmid (Promega), excised with BamH-I and Hind-III restriction enzymes and then subcloned in pET28a expression plasmid. Protein expression was carried out in *E. coli* (BL21 DE3 strain) system by IPTG induction followed by purification with Ni^2+^-NTA Agarose His-tagged protein purification system (QIAGEN). The expressed recombinant protein (~15 kDa) was sequenced for identity confirmation by mass spectrometry at the protein sequencing platform at FIOCRUZ.

### LlTGF-β Anti-serum Production

LlTGF-β*rec* (1 mg) was inoculated into 45 days old New Zealand male rabbit with Freund's complete adjuvant, with two subsequent boosts using incomplete Freund's adjuvant. Animal use was approved by the Instituto Oswaldo Cruz Ethical Commitee on Animal Use (CEUA/IOC-010/2018). Serum titration was carried out by immuno-dot blot (not shown), using the corresponding antigen, and revealed with horseradish peroxydase (HRP)-labeled goat anti-rabbit IgG as the secondary antibody. Western blot assays were also carried to verify LlTGF-β anti-serum specificity in sand fly dissected gut samples (Figure S2).

### ELISA

ELISA high binding microplates (NOH® ELISA Plate—LBEP196) were coated overnight in a moist chamber with the protein extract of 12 midguts from *L. longipalpis* 72 h after feeding on blood or blood containing *L. i. chagasi*. 5 μg of LlTGF-β*rec* was also used as a positive control. Wells were blocked overnight at 4°C with 1% BSA in PBS. On the following day, wells were incubated overnight at 4°C with anti-LlTGF-β serum 1:100 diluted in 1% BSA in PBS. Wells were washed three times with PBS and incubated overnight at 4°C with 1:40.000 dilution of goat anti-rabbit IgG peroxidase-conjugated antibody. After washing, plates were revealed by adding to each well 50 μl of 3,3′,5,5′-Tetramethylbenzidine (TMP) substrate solution followed by a 15 min incubation at room temperature. Following this step, 50 μl of stop solution (0.2 M H_2_SO_4_) was added to each well and incubated for 30 min. The endpoint absorbance was measured at 450 nm.

### Nitrite Detection in Sand Fly Guts

Detection of Nitrite-derived NO was performed with pools of 10 midguts (with Malpighian tubules removed) dissected from *LlTGF*-β or β-gal (control group) dsRNA injected flies artificially fed on defibrinated rabbit blood, or non-injected insects fed on blood containing 10 μM of the TGF-β receptor inhibitor SB431542 or DMSO (control group). Samples were collected at 24, 48, and 72 h after feeding, homogenized in 0.9% NaCl solution, and centrifuged. The supernatant was split in 2 replicates of 50 μL, loaded in a clear polystyrene medium-binding flat bottom 96-well plate (Corning, SIGMA) for Griess Reagent nitrite detection in 150 μL final volume assay following standard procedures. Nitrite levels were measured under 550 nm wave-length in an Infinite M2000 plate reader (TECAN, Switzerland).

## Results

### *LlTGF-β* Sequence and Phylogenetic Tree

The full *LlTGF*-β sequence (GenBank: MF074142) contains 1,158 nucleotides that encode a 386 amino acid sequence (Figure S1). The amino acid sequence contains eight conserved cysteine residues involved in disulfide bonds and a RXXR motif that is a putative catalytic domain. The TGF-β superfamily domain was identified from amino acids 280 to 385.

**Figure 1:**
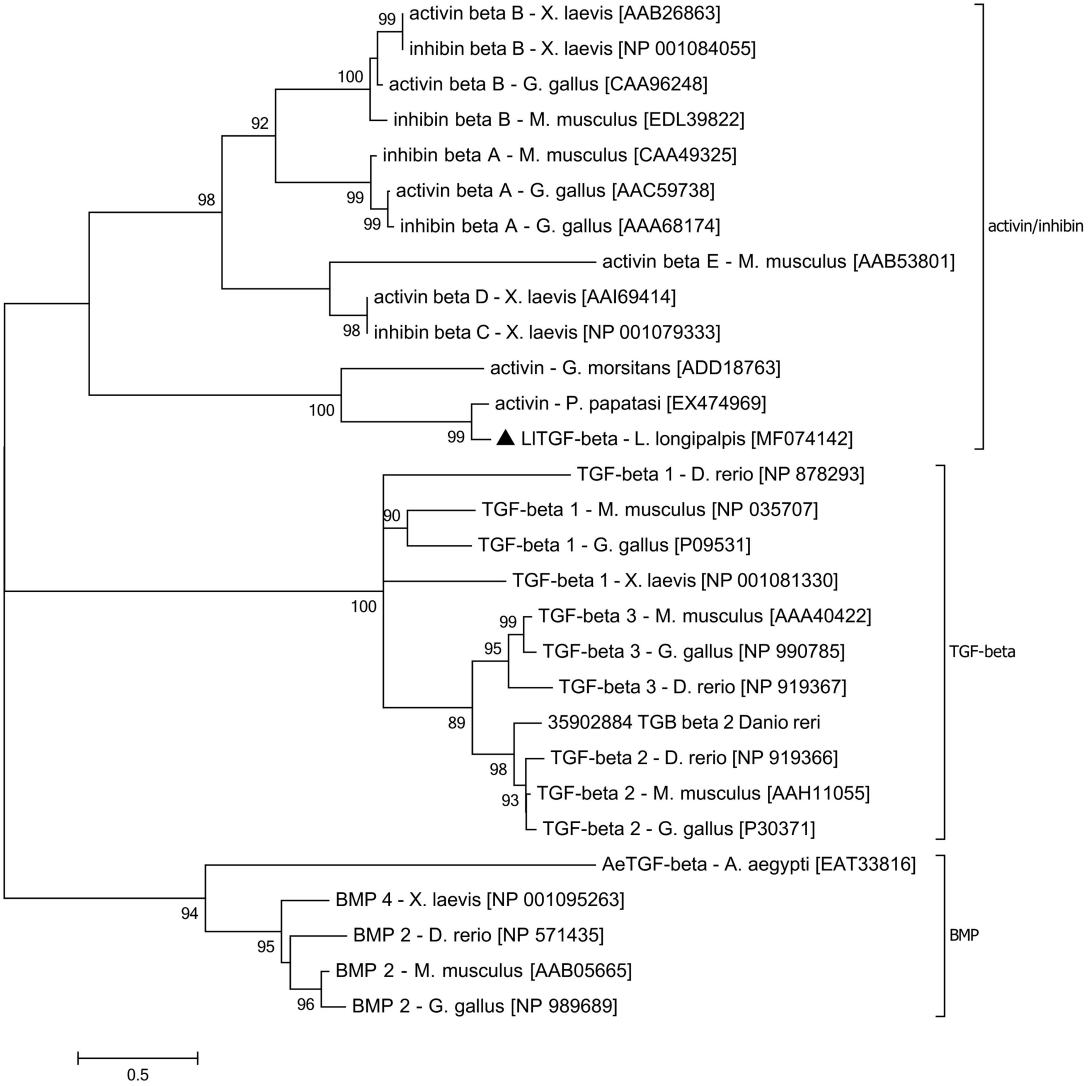
LlTGF-β phylogenetic tree. The LlTGF-β deduced amino acid sequence was aligned with other TGF-β sequences from the insect vectors *Aedes aegypti, Glossina morsitans, Phlebotomus papatasi*, and the vertebrates *Danio rerio, Gallus gallus, Mus musculus, Xenopus laevis*. The evolutionary history was inferred using MEGA 6.06 software with Maximum Likelihood method based on the JTT matrix-based model. Evolutionary rate differences among sites [5 categories (+G, parameter = 3.2942)] were modeled by discrete Gamma distribution. The rate variation model allowed for some sites to be evolutionarily invariable ([+I], 2.1884% sites). Positions with gaps and missing data were eliminated. The TGF-β subfamily groups are indicated on the right side of the phylogram. Each sequence is labeled with species name followed by GenBank accession number. Branch length represents numbers of substitutions per site. Numbers on the tree nodes indicate bootstrap values higher than 85% (500 replicates).

TGF-β family aminoacid sequences available on NCBI data base were aligned in order to create a TGF-β family tree and assess subgroup similarities. Sequences obtained from organisms of different taxa but belonging to the same TGF-β subfamily were grouped such as activin/inhibin, TGF-β *sensu stricto*, and glass bottom boat (GBB) member of the BMP subfamily (Figure 1). *LlTGF*-β grouped with *G. morsitans* and *P. papatasi* activin/inhibin sequences with high bootstrap support.

### *LlTGF-β* Gene Expression in Immature and Adult Insects, and in Infected Females

**Figure 2:**
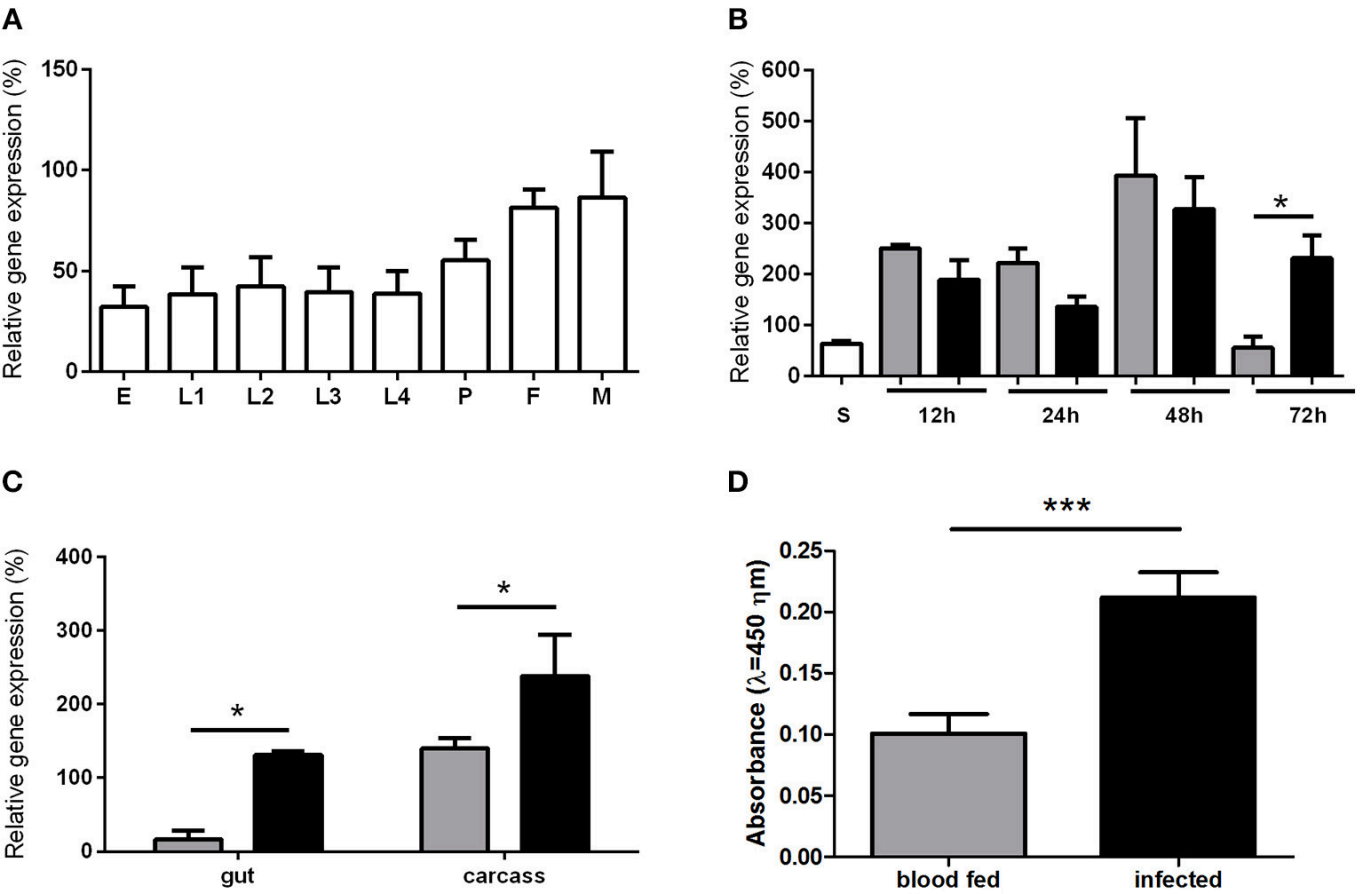
*LlTGF*-β gene expression in sand flies. **(A)** *L. longipalpis* development stages: egg (E), larval stages (L1, L2, L3, L4), pupae (P), adult female (F), and male (M). **(B)** *L. longipalpis* fed on sucrose (S) are represented by white bar. Flies collected at 12, 24, 48, and 72 h post blood meal are represented by gray bars or after feeding on infective blood represented by black bars. **(C)** *LlTGF*-β expression in gut and carcass 72 h after ingestion of blood without (gray bars) or with (black bars) *Leishmania*. Gene expression by semi-quantitative PCR was calculated relative to histone or RP49 *L. longipalpis* reference genes. **(D)** ELISA for detection of LlTGF-β protein 72 h after blood or infective meal. Bars represent mean with standard error of 3 replicates. Significant differences were evaluated by *t*-test and Mann-Whitney post-test (*p < 0.05; ***p < 0.001).

Due to the activin/inhibin subfamily association to growth and development regulation we investigated its expression in sand fly larval and adult stages. The *LlTGF*-β gene was ubiquitously expressed in all *L. longipalpis* developmental stages (Figure 2A), with a slight non-significant increase in adult male and female insects. Comparison among sugar fed, blood fed and *Leishmania* infected sand flies females revealed that blood ingestion induced *LlTGF*-β expression when compared to sugar fed females (Figure 2B). Additionally, *LlTGF*-β gene was significantly upregulated in *Leishmania* infected females when compared to blood fed insects at 72 h post feeding (Figure 2B). This increased *LlTGF*-β transcription occurred in both carcass and dissected gut (Figure 2C). Our attempts to use Western blot to verify the presence of LlTGF-β in *L. longipalpis* guts were hindered by the recognition of mammal TGF-β in blood fed insects (Figure S2). Nevertheless, these bands disappeared rapidly and were totally absent at 24, 48, and 72 h post-blood-feeding. Using the more sensitive ELISA detection we also observed an increased production of LlTGF-β at the protein level in *Leishmania* infected compared to blood fed females at 72 h after feeding (Figure 2D).

### *Leishmania* Infection of Sandflies Silenced for LlTGF-β or Fed With TGF-β Receptor Inhibitor SB431542

**Figure 3:**
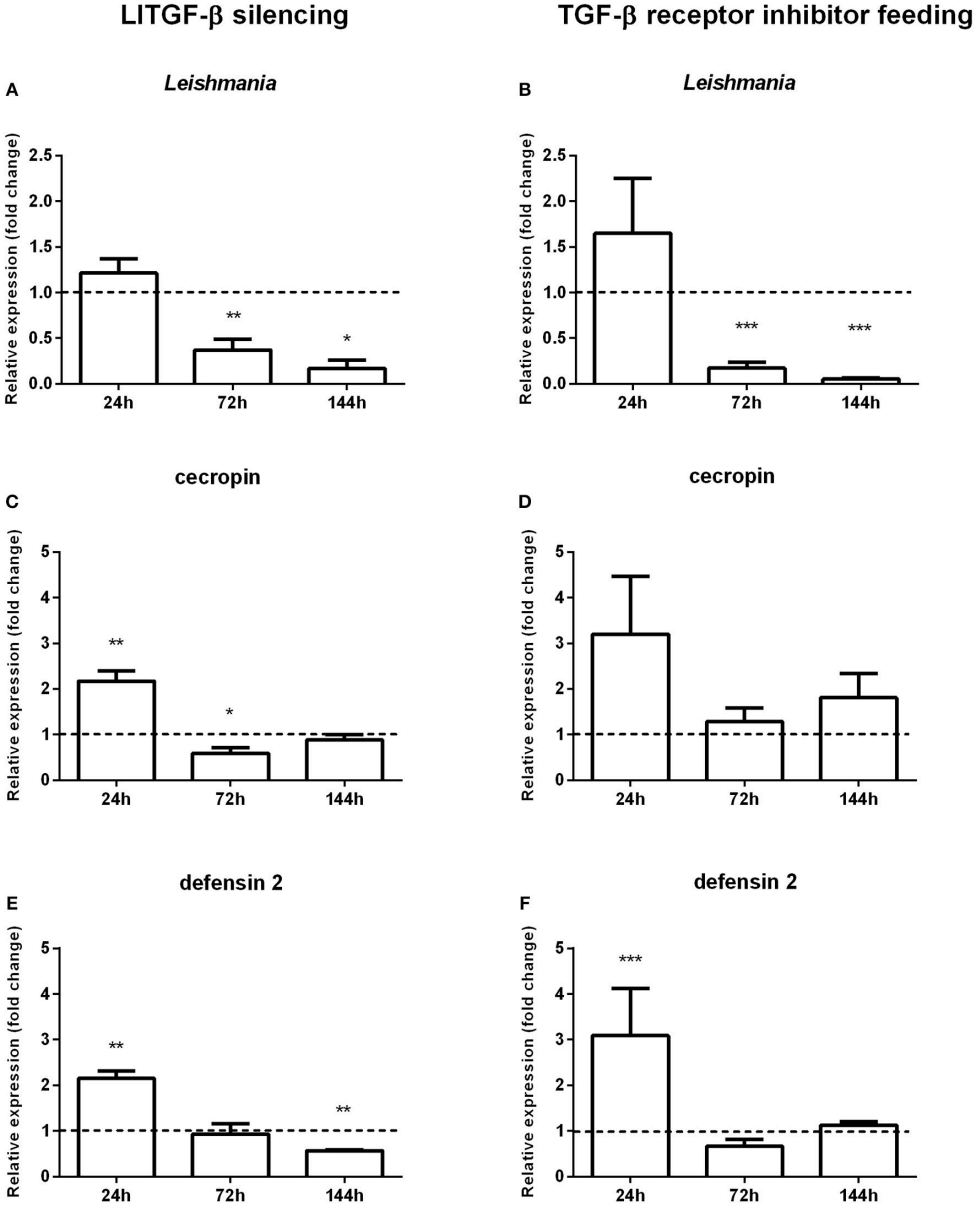
Gene expression in sand flies infected by *L. i. chagasi* after abrogation of the LlTGF-β signaling pathway. **(A,C,E)** Gene expression of LlTGF-β silenced insects. **(B,D,F)** Gene expression of insects fed with TGF-β receptor inhibitor. **(A,B)** Quantification of *L. i. chagasi* in sand flies. **(C,D)** Relative expression of cecropin. **(E,F)** Relative expression of defensin 2. Samples were collected at 24, 72, and 144 h post infection. Dotted lines indicate gene expression of β-galactosidase dsRNA injected **(A,C,E)** or DMSO fed flies **(B,D,F)** control groups, or. Both test and control groups were infected by *L. i. chagasi*. Comparisons were done between silenced vs. non-silenced, or inhibitor treated vs. non-treated sand fly groups. Bars represent mean with standard error of fold change in gene expression relative to control groups of 3 independent experiments. Significant differences were evaluated by *t*-test and Mann–Whitney post-test (*p < 0.05; **p < 0.01; ***p < 0.001).

To investigate the involvement of the TGF-β signal transduction pathway on the evolution of *Leishmania* infection in *L. longipalpis* this pathway was abrogated using RNAi to silence *LlTGF*-β (Figure S3A) or employing a TGF-β receptor inhibitor. After each treatment *L. longipalpis* were infected with *L. i. chagasi*. Both approaches had as consequence a significant decrease in *Leishmania* infection at 72 and 144 h post infection (Figures 3A,B).

### Gene Expression of Immune Effectors After LlTGF-β Knock-Down or Feeding With TGF-β Receptor Inhibitor SB431542 Followed by *Leishmania* Infection

The decreased *L. longipalpis* infection by *Leishmania* upon the TGF-β signaling pathway inhibition led us to investigate the expression of some effector molecules. We assessed the effect of *LlTGF*-β silencing or TGF-β receptor inhibitor treatment on Toll regulated AMPs, such as defensin 2 and cecropin. In sand flies with TGF-beta silenced and infected with *L. i. chagasi* a statistically significant increase of both cecropin (Figure 3C) and defensin 2 (Figures 3E,F) expression was observed only at 24 h post infection, with a significant decrease at 72 and 144, respectively. In sand flies fed with the TGF-β receptor inhibitor and infected with *Leishmania*, cecropin expression levels increased at 24 h, although not significantly (Figure 3D).

### Regulation of NO Production by LlTGF-β

**Figure 4:**
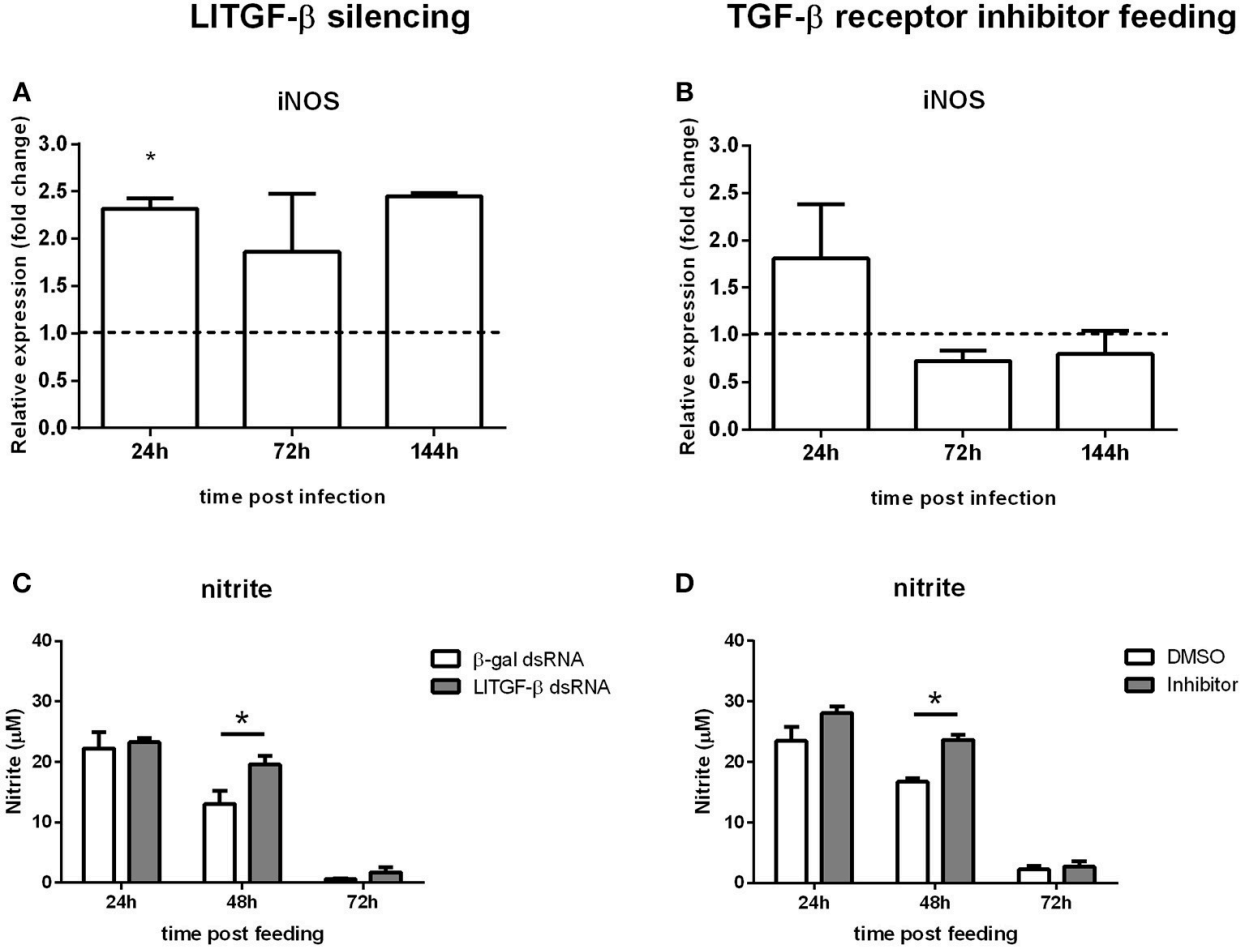
Nitric oxide production in sand flies abrogated for the LlTGF-β signaling pathway. **(A)** iNOS gene expression of LlTGF-β silenced insects, **(B)** fed with blood containing TGF-β receptor inhibitor, both **(A,B)** infected by *L. i. chagasi*. Samples were collected at 24, 72, and 144 h post infection. Dotted line indicates gene expression of control group, β-gal dsRNA injected or blood containing DMSO fed flies. Bars represent mean with standard error of fold change in gene expression relative to control groups of three independent experiments. Significant differences were evaluated by *t*-test and Mann-Whitney post-test. **(C)** NO measurement in LlTGF-β silenced sand flies fed on blood. **(D)** Nitrite measurement in flies fed on blood containing TGF-β receptor inhibitor. Bars represent mean with standard error of nitrite measurements in test and control groups of 3 independent experiments. Significant differences were evaluated by *t*-test and Mann-Whitney post-test (*p < 0.05).

To test the possible role of LlTGF-β transduction signaling pathway on NO production, we assessed iNOS expression by qPCR. iNOS levels were increased in LlTGF-β silenced insects at 24 h post infection (Figure 4A) and presented a slight but not significant increase on TGF-β receptor inhibitor SB431542 fed insects at 24 h post infection (Figure 4B). We tested the nitrite production levels in LlTGF-β silenced insects. In non-fed silenced insects nitrite levels were very low and no detectable modulation occurred (Figure S3B). Since the insect acquire *Leishmania* parasites through blood feeding we also measured the nitrite levels after LlTGF-β gene silencing or TGF-β receptor inhibitor treatements on blood fed *L. longipalpis*. LlTGF-β gene silencing was followed by an increase of nitrite levels in sand fly guts at 48 h post blood feeding when compared to control groups (Figure 4C). A similar result was obtained in TGF-β receptor inhibitor treated females, with an increase of nitrite at 48 h post blood feeding (Figure 4D).

## Discussion

TGF-β belongs to a family of multifunctional cytokines found in organisms that go from arthropods to mammals (Massague, 1990). TGF-β molecules belonging to different subfamilies has been identified in many insect species including *Bombyx mori* (Hu et al., 2016), *Drosophila* (reviewed in Hinck et al., 2016), and the mosquitoes *Culex pipiens* (Hickner et al., 2015) and *Anopheles stephensi* (Crampton and Luckhart, 2001). In the malaria vector *A. stephensi*, a TGF-β homolog named As60A was implicated in the insect immune response to *Plasmodium* (Crampton and Luckhart, 2001). It was also determined that the mammalian TGF-β 1 present in the ingested blood regulates NO production, modulating the *A. stephensi* immune response against the parasite (Luckhart et al., 2003).

In sand flies, this is the first report on the characterization of a TGF-β family gene. The identified *LlTGF*-β sequence contains the catalytic and conserved signature domains of the TGF-β superfamily. Phylogenetic analysis showed LlTGF-β to be highly similar to the activin/inhibin subfamily.

In insects, as much as in other organisms, TGF-β family molecules are involved in ontogeny (O'Connor et al., 2006). The expression profile of the *L. longipalpis* TGF-β gene shows that it is expressed in all developmental stages and may thus be involved in sand fly ontogeny as well. Additionaly, members of the TGF-β family have a role in nutrient detection and therefore may regulate nutrient acquisition (Chng et al., 2014). In the case of *L. longipalpis LlTGF*-β expression in blood fed females was increased after blood intake, indicating that the presence of nutrients stimulates activin production. Regarding the effect of *L. longipalpis* infection with *Leishmania*, LlTGF-β expression was increased at 3 days post infection, when parasites are in close contact with the insect midgut epithelium, shown both by RT-PCR as well as by ELISA. This is in line with the fact that the activin/inhibin subfamily may be involved in immune response.

TGF-β is considered an anti-inflammatory and immunosuppressive cytokine in mammals. When mice chronically infected with *L. major* were treated with an antibody anti-TGF-β a pro-inflammatory environment was created with consequent decrease of parasite numbers at the lesion site (Li et al., 2018). In addition, *in vitro* assays showed that the incubation of recombinant TGF-β with *L. braziliensis* infected macrophages increased parasite loads (Barral et al., 1995). In an inflammatory situation, Toll-like receptors (TLRs) mediate the activation of activins that consequently suppresses the immune response (negative feedback; Sideras et al., 2013).

To investigate the putative participation of the insect LlTGF-β signaling pathway in response to parasitic infection, we blocked this signaling pathway. Both blocking gene translation or protein interaction with a receptor generated a strong decrease on parasite numbers in late time points, revealing that the suppression of this cytokine-like has a deleterious effect on the parasite. This indicates that this molecule most probably has an anti-inflammatory role in *L. longipalpis*, as already seen in mammals.

We assessed the effects of suppressing the TGF-β signaling on sand fly immune effector molecules. The silencing of *LlTGF*-β or inhibition of a TGF-β receptor resulted in an early increased expression of the AMPs cecropin and defensin suggesting that the *L. longipalpis* activin-like molecule can have a suppressing effect over AMPs expression. AMPs production is one of the main responses against infection in insects and is regulated mainly by the Toll and IMD pathways (Leulier et al., 2003; Kaneko et al., 2004). In *Drosophila*, AMPs are crucial for the maintenance of gut homeostasis, especially in what concerns microbiota regulation (Bosco-Drayon et al., 2012), and the *dpp* and *dawdle*, members of the TGF-β family, have a suppressing effect on AMPs expression (Coggins et al., 2012).

Besides the repressor effect on the insect immunity, activins were previously shown to be involved in immune response regulation throught other pathways. Studies with parasitic infections in vertebrates showed that TGF-β family members are responsible not only for regulating initial pro-inflammatory and late anti-inflammatory responses (Vodovotz et al., 2004), but are also involved in repressing inducible nitric oxide synthase (iNOS) expression, stability and activity in different cell types through mechanisms that were not fully understood (Berg et al., 2007; Aleman-Muench and Soldevila, 2012).

In *A. stephensi*, the TGF-β gene As60A expression was induced by infection with *Plasmodium berghei* (Crampton and Luckhart, 2001). Interestingly, human blood derived TGF-β was able to activate the mosquito TGF-β pathway leading to the activation of the MAPK/ERK cascade and downregulation of NO production (Surachetpong et al., 2009). Both *A. stephensi* and *Drosophila* are able to modulate ROS levels to combat pathogen infections (Luckhart et al., 1998; Ha et al., 2005; Shrinet et al., 2014). On the other hand, NO mediated an early *Drosophila* innate immune responses through diptericin and drosomycin expression (Foley and O'Farrell, 2003) indicating that it is an important signaling molecule.

ROS production in *L. longipalpis* immune response against two different pathogens, *Leishmania mexicana* and *Serratia marcescens*, elicited different immune responses. *S. marcescens* infection increased ROS levels in the insect gut whereas *L. mexicana* did not (Diaz-Albiter et al., 2012). The RNAi gene silencing of *L. longipalpis* catalase, a ROS-detoxifying gene, led to decreased *L. mexicana* infection. These results suggest that ROS are harmful to *Leishmania* and *S. marcescens* but *Leishmania* is able to modulate the *L. longipalpis* oxidative stress response while *S. marcescens* is not (Diaz-Albiter et al., 2012).

Our results showed that blocking the TGF-β pathway increased iNOS expression in early times post *Leishmania* infection. In the following days infection levels were reduced indicating that, in the absence of an intact TGF-β pathway, increased iNOS levels can be deleterious to *Leishmania*. We would expect that under normal LlTGF-β conditions it would control iNOS expression and consequently nitrite levels. To test this hypothesis we measured nitrite levels in blood fed flies under the effect of both TGF-β pathway blockage methods. We observed that there was a significant increase of nitrite levels 2 days post blood feeding. In hematophagous insects blood digestion requires an efficient control of oxidative stress (Souza et al., 1997) and in *L. longipalpis* the nitrite levels in blood fed gut reached nearly 100 times higher than sugar fed insects. In order to control the harmful effects of these free radicals insects have a plethora of mechanisms to keep the ROS balance in the gut. In *L. longipalpis* the TGF-β blockade significantly hindered the nitrite balance in the gut on the second day of blood feeding, time when the subproducts of blood digestion are released in high levels (Graça-Souza et al., 2006). High doses of NO are implicated in parasite killing, but in the case of *Leishmania* infection the parasites proliferate in the insect gut apparently benefitting from LlTGF-β controlling NO levels.

Our results show the conservation of the TGF-β-mediated signaling in the presence of *Leishmania* infection in vector and vertebrate hosts, and a role for this citokine-like molecule in the regulation of AMPs expression and nitrite levels.

## Data Availability

The datasets generated for this study can be found in GenBank, MF074142.

## Author Contributions

TD-B, ET, AT, YT-C contributed conception and design of the study; TD-B, ET, CM, RMC, MS-N, MJ performed experiments; TD-B, ET, AT, YT-C participated in manuscript writing; PV contributed with reagents. All authors contributed to manuscript revision, read and approved the submitted version.

### Conflict of Interest Statement

The authors declare that the research was conducted in the absence of any commercial or financial relationships that could be construed as a potential conflict of interest.

## Supplementary Material

Figure S1 *LlTGF*-β cDNA and deduced amino acid sequences. Numbers on the left side indicate nucleotides. Numbers on right indicate amino acid residues. Cleavage signal peptide site is indicated by a black triangle (between residues 24 and 25). Conserved cysteine residues are shown in bold characters (residues 282, 283, 311, 315, 350, 351, 383, and 385). Putative catalytic domain motif is indicted by a rectangle (residues 268 to 271). The TGF-β superfamily domain is indicated by underlined characters (residues 280 to 385).

Figure S2 LlTGF-β antiserum assay detecting a single polypeptide from *L. longipalpis* gut samples. Samples corresponding to 2 guts dissected at 0 (non-fed), 2, 6, 12, 24, 48, and 72 h after blood feeding, and recombinant LlTGF-β as positive control (750 ng) were separated by 12% SDS-PAGE under constant 100 V, and transferred to nitrocellulose membrane during 1 h at 4°C. Membranes were blocked with 5% low-fat dried milk in Tris buffered saline (TBS) supplemented with 0.05% Tween-20. Membranes were washed three times with TBS and incubated for 1 h with anti-LlTGF-β serum at 1:500 dilution. HRP-conjugated goat anti-rabbit IgG at a 1:40,000 dilution was used as secondary antibody. The relative molecular mass of the reactive polypeptides was calculated by comparison with the mobility of molecular mass standards, using ImageJ 1.42q software (NIH, USA).

Figure S3 LlTGF-β silencing: Non-blood fed flies were injected with LlTGF-β dsRNA or β-gal dsRNA (control group) and kept with sugar meal *ad libitum*. **(A)** LlTGF-β gene expression in non-blood fed females after LlTGF-β dsRNA microinjection is expressed relative to control group indicated by dotted line as described previously in manuscript qPCR methods; **(B)** Guts were dissected at 24, 48, and 72 h pos dsRNA injection for nitrite detection assays. Gray bars represent nitrite levels in LlTGF-β dsRNA injected insects. White bars represent nitrite levels in β-gal dsRNA injected insects. Graphics represent mean with standard error of 3 independent experiments. Significant differences were evaluated by *t*-test and Mann-Whitney post-test (^*^*p* < 0.05; ^**^*p* < 0.01).
