# Supplementary figures and images for: Lutzomyia longipalpis TGF-β Has a Role in Leishmania infantum chagasi Survival in the Vector
Source: Front Cell Infect Microbiol. 2019 Mar 27;9:71. doi: 10.3389/fcimb.2019.00071 (PMC6445956; doi:10.3389/fcimb.2019.00071)

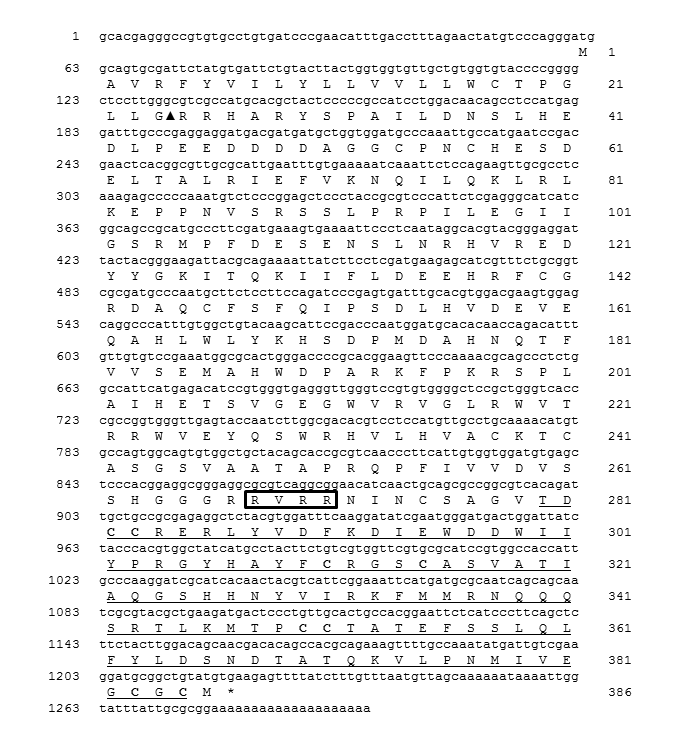

Supplement: Figure S1 — LlTGF-β cDNA and deduced amino acid sequences. Numbers on the left side indicate nucleotides. Numbers on right indicate amino acid residues. Cleavage signal peptide site is indicated by a black triangle (between residues 24 and 25). Conserved cysteine residues are shown in bold characters (residues 282, 283, 311, 315, 350, 351, 383, and 385). Putative catalytic domain motif is indicted by a rectangle (residues 268 to 271). The TGF-β superfamily domain is indicated by underlined characters (residues 280 to 385). [file Image_1.TIF]

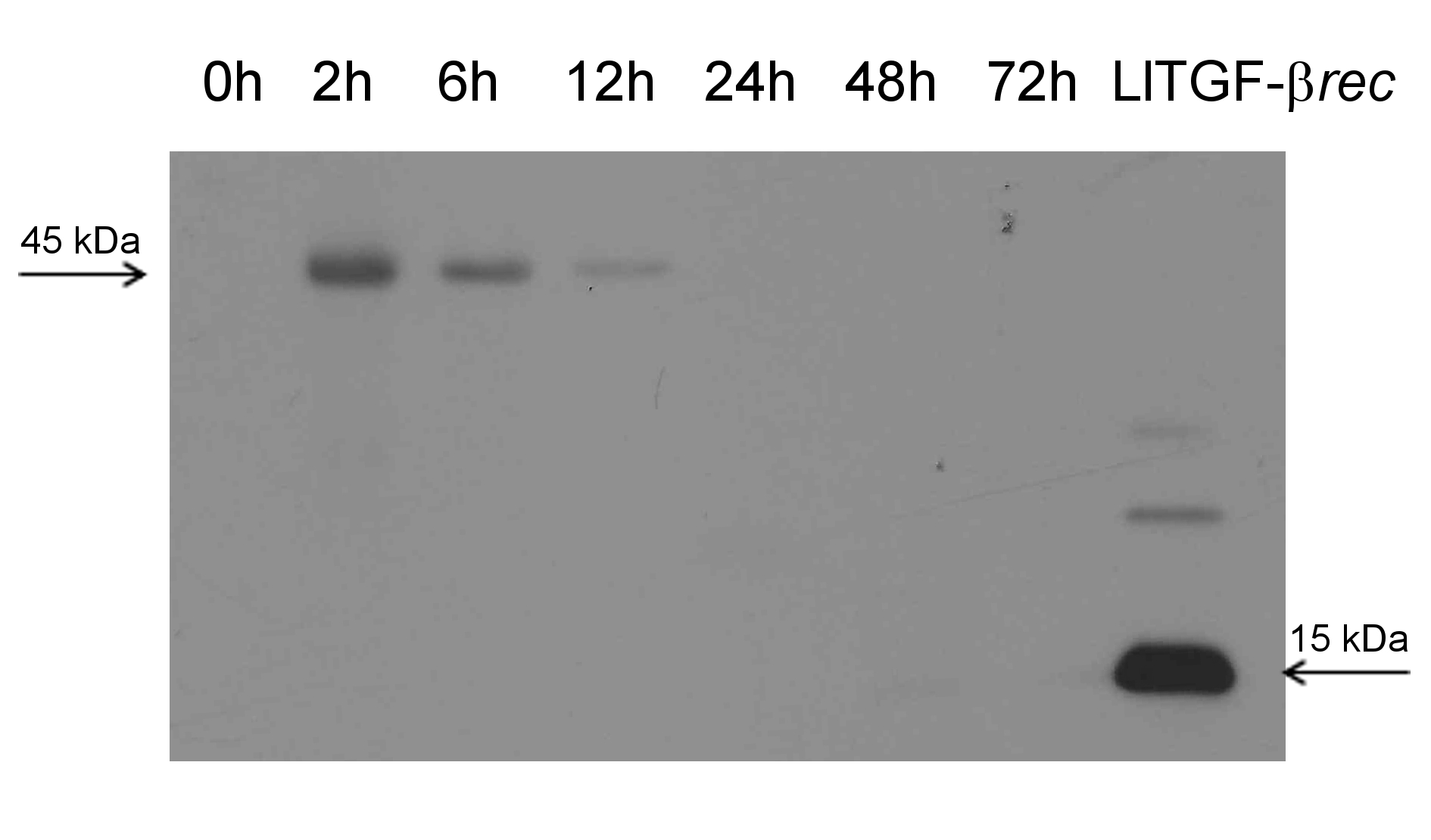

Supplement: Figure S2 — LlTGF-β antiserum assay detecting a single polypeptide from L. longipalpis gut samples. Samples corresponding to 2 guts dissected at 0 (non-fed), 2, 6, 12, 24, 48, and 72 h after blood feeding, and recombinant LlTGF-β as positive control (750 ng) were separated by 12% SDS-PAGE under constant 100 V, and transferred to nitrocellulose membrane during 1 h at 4°C. Membranes were blocked with 5% low-fat dried milk in Tris buffered saline (TBS) supplemented with 0.05% Tween-20. Membranes were washed three times with TBS and incubated for 1 h with anti-LlTGF-β serum at 1:500 dilution. HRP-conjugated goat anti-rabbit IgG at a 1:40,000 dilution was used as secondary antibody. The relative molecular mass of the reactive polypeptides was calculated by comparison with the mobility of molecular mass standards, using ImageJ 1.42q software (NIH, USA). [file Image_2.JPEG]

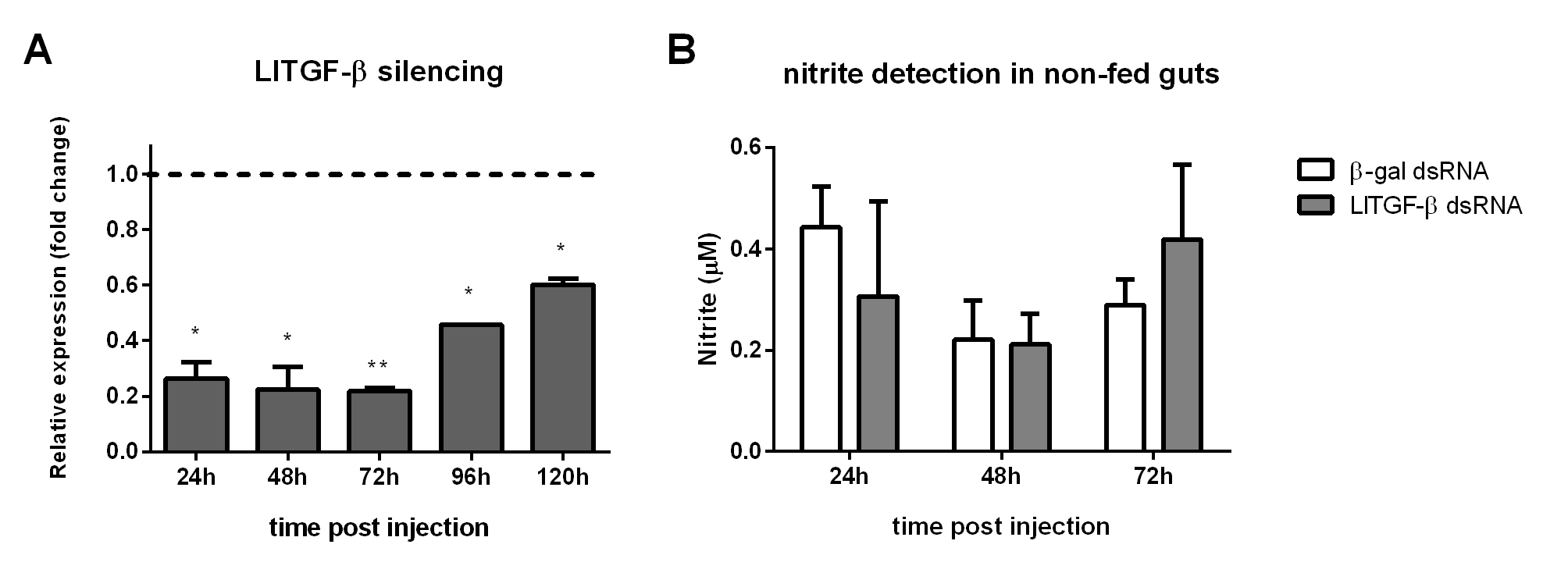

Supplement: Figure S3 — LlTGF-β silencing: Non-blood fed flies were injected with LlTGF-β dsRNA or β-gal dsRNA (control group) and kept with sugar meal ad libitum. (A) LlTGF-β gene expression in non-blood fed females after LlTGF-β dsRNA microinjection is expressed relative to control group indicated by dotted line as described previously in manuscript qPCR methods; (B) Guts were dissected at 24, 48, and 72 h pos dsRNA injection for nitrite detection assays. Gray bars represent nitrite levels in LlTGF-β dsRNA injected insects. White bars represent nitrite levels in β-gal dsRNA injected insects. Graphics represent mean with standard error of 3 independent experiments. Significant differences were evaluated by t-test and Mann-Whitney post-test (*p < 0.05; **p < 0.01). [file Image_3.JPEG]
